# The complete chloroplast genome of Zelkova schneideriana (Rosales: Ulmaceae), an Endangered species endemic to China

**DOI:** 10.1080/23802359.2018.1483762

**Published:** 2018-07-27

**Authors:** Chao Yang, Xiao-Long Ren, Gui-Liang Xin, Zonglin Lei, Xiaomin Du, Huidong Liu, Wen-Zhe Liu

**Affiliations:** Key Laboratory of Resource Biology and Biotechnology in Western China (Northwest University), School of Life Science, Ministry of Education, Northwest University, Xi’an, China

**Keywords:** *Zelkova schneideriana*, Endangered species, chloroplast genome, phylogenetic analysis

## Abstract

*Zelkova schneideriana* Hand.-Mazz. (Ulmaceae) is an endangered species endemic to China. In this study, we reported its complete chloroplast (cp) genome based on Illumina pair-end sequencing. The whole genome was 158,999 bp long, consisting of a pair of inverted repeat (IR) regions of 26,427 bp, a large single copy (LSC) region of 87,397 bp and a small single copy (SSC) region of 18,748 bp. The cp genome contained 133 genes, including 88 protein-coding genes (80 PCG species), 37 tRNA genes (30 tRNA species), and eight rRNA genes (4 rRNA species). The overall G+C content of the whole genome was 35.6%, and the corresponding values of the LSC, SSC, and IR regions were 33.0, 28.3, and 42.4%, respectively. The maximum likelihood phylogenetic analysis of 25 selected chloroplast genomes demonstrated that *Z*. *schneideriana* was closely related to *Ulmus macrocarpa* and *Ulmus pumila*.

*Zelkova schneideriana* Hand.-Mazz., belonging to family Ulmaceae, is mainly distributed in Huai River Basin, Qinling Mountains, and the Lower-middle Reaches of the Yangtze River in China (Zhang et al. 2013). As a consequence of the excessively cut for utilizing, this rare species endemic to China is under the threat of extinction and has been classified as a vulnerably Endangered plant listed into Chinese Dangerous Plant Red Paper (Class II) (Fu 1992). Therefore, there is immediate need to take effective measures to protect this species. Here, we characterized the complete chloroplast (cp) genome of *Z*. *schneideriana* based on high-throughput Illumina sequencing technology, which will contribute to the further studies on the genetic diversity, systematic evolution, and conservation of this Endangered tree species. The annotated cp genome of *Z*. *schneideriana* has been deposited into GenBank with the accession number MG717940.

Fresh leaves of a *Z*. *schneideriana* individual were collected from Fujian Agriculture and Forestry University (Fujian, China; 26°03′N, 119°16′E). A shotgun library was constructed and whole genome sequencing was performed with 150 bp pair-end (PE) reads on the Illumina Hiseq 2500 platform (Illumina, CA). In total, 30,214,272 raw reads of *Z*. *schneideriana* were obtained and their qualities were filtered and trimmed using NGS QC Toolkit v2.3.3 (Patel and Jain 2012). Then, 144,092 clean reads were assembled by MITObim v1.8 (Hahn et al. 2013) with the sequences of published *Ulmus davidiana* (KY244082) cp genomes as the reference. The cp genome sequence was annotated using the program Geneious R8.0.2 (Biomatters Ltd., Auckland, New Zealand).

The complete cp genome of *Z*. *schneideriana* was 158,999 bp in length, containing a pair of inverted repeat (IR) regions of 26,427 bp which were separated by a large single copy (LSC) region of 87,397 bp and a small single copy (SSC) region of 18,748 bp. It encoded 133 genes, including 88 protein-coding genes (80 PCG species), 37 transfer RNA genes (30 tRNA species), and eight ribosomal RNA genes (four RNA species). Among these genes, a total of 19 gene species had two copies in the IR regions, including eight PCG species (*rps19*, *rpl2*, *rpl23*, *ycf2*, *ycf15*, *ndhB*, *rps7*, and *ycf1*), seven tRNA species (*trnI-CAU*, *trnL-CAA*, *trnV-GAC*, *trnI-GAU*, *trnA-UGC*, *trnR-ACG*, and *trnN-GUU*), and four rRNA species (*rrn16*, *rrn23*, *rrn4.5*, and *rrn5*). Nine PCG species (*rps16*, *rpoC1*, *rpl12*, *petB*, *petD*, *rpl16*, *rpl2*, *ndhB*, and *ndhA*) and six tRNA species (*trnK-UUU*, *trnG-GCC*, *trnL-UAA*, *trnV-UAC*, *trnI-GAU*, and *trnA-UGC*) possessed a single intron, while another three gene species (*rps12*, *ycf3*, and *clpP*) contained two introns. The overall GC content of the cp genome is 35.6%, with the corresponding values of 33.0, 28.3, and 42.4% for the LSC, SSC, and IR regions, respectively.

To ascertain its phylogenetic position within the order Rosales, a maximum likelihood (ML) tree was reconstructed using the GTR + G + I model, with 1000 bootstrap replicates, in RAxML v.8.2.8 (Stamatakis 2014). The phylogenetic analysis indicated that *Z*. *schneideriana* was closely related to *Ulmus macrocarpa* and *Ulmus pumila* (Figure 1).

**Figure 1.**
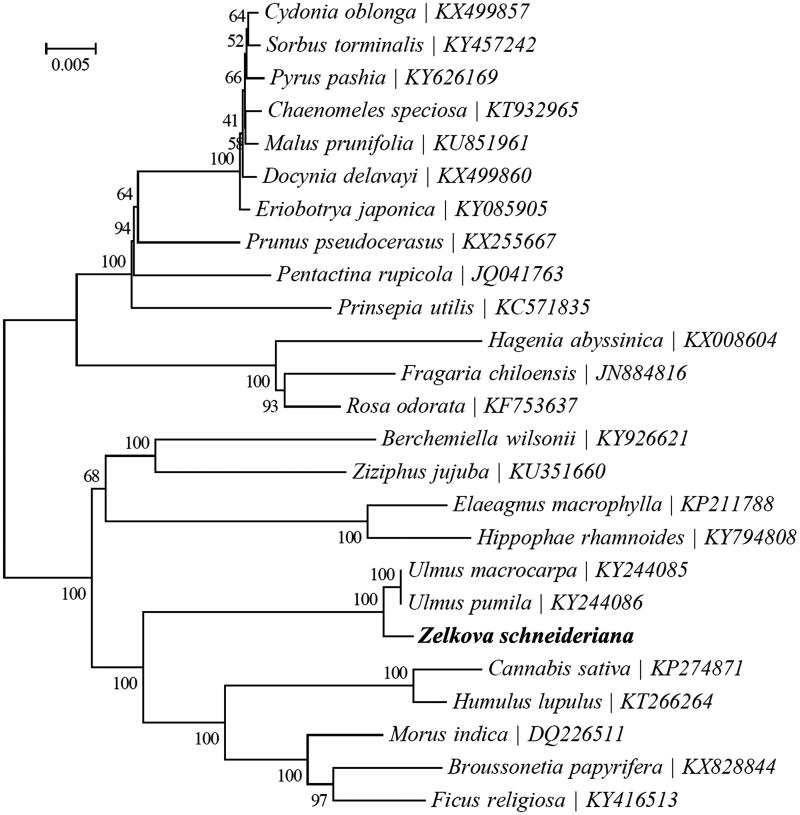
Maximum likelihood phylogenetic tree based on 25 complete chloroplast genome sequences.
